# Home-Visit Intervention to Reduce Stress of Underserved Family Caregivers for Persons With Dementia

**DOI:** 10.1093/geroni/igab046.585

**Published:** 2021-12-17

**Authors:** Jung-Ah Lee, Seyed Amir Hossein Aqajari, Eunae Ju, Priscilla Kehoe, Lisa Gibbs, Amir Rahmani

**Affiliations:** 1 University of California, Irvine, Irvine, California, United States; 2 University of California Irvine, Irvine, California, United States; 3 UC Irvine Health, UC Irvine Health, California, United States

## Abstract

Immigrant family caregivers for persons living with dementia (PWD) have constant stress due to the 24/7 responsibility. These family caregivers of PWD often have high morbidity and mortality. We provided a cultural and language specific home-visit intervention for these vulnerable family caregivers. There is a lack of an objective measure of stress for caregivers. We assessed caregivers’ stress by measuring heart rate variability (HRV), a physiological measure of stress, using a smartwatch for a one-month intervention. Weekly home visits for a month were provided to dementia family caregivers by trained community health workers with stress reduction techniques: mindful breathing and compassionate listening. Linear mixed-effect models were used to analyze the trends for the daily stress levels as measured by HRV from the smartwatch. We had 22 participants who completed the 4-week intervention (8 Latinos, 8 Koreans, 6 Vietnamese). The models showed a significant decrease in the stress level of all participants for 3 weeks (all Ps<0.01). At 28 days (4 weeks) all three groups showed a decrease in stress: Korean group (Beta= -0.405, P<0.001), Vietnamese group (Beta = -0.150, P=0.028), Latino group (Beta= -0.154, P=0.073) and all caregivers (Beta = -0.235, P< 0.001). The findings demonstrated a reduction of immigrant family caregiver stress with a home-visit weekly intervention for one month using mindful breathing and compassionate listening by culturally/linguistically appropriate community health workers. Large-scale studies to determine long-term outcomes of family dementia caregivers are necessary and should be carried out.

